# Protoplast isolation from *Dictyopteris pacifica* and *Scytosiphon lomentaria*, using a simple commercial enzyme preparation

**DOI:** 10.1186/s43141-021-00226-y

**Published:** 2021-09-03

**Authors:** Jose Avila-Peltroche, Boo Yeon Won, Tae Oh Cho

**Affiliations:** 1 Department of Life Science, Chosun University, Gwangju, 61452 Korea; 2 Department of Integrative Biological Sciences & BK21 FOUR Educational Research Group for Age-associated Disorder Control Technology, Chosun University, Gwangju, 61452 Korea

**Keywords:** Brown algae, Commercial enzymes, *Dictyopteris pacifica*, *Scytosiphon lomentaria*, Protoplast isolation, Cultured samples

## Abstract

**Background:**

Protoplasts (i.e., naked plant cells) can be used for in vitro manipulations and genetic improvement in cultivars with economic value. During the last decade, protoplast research in economic brown algae has been scarce, and it is usually hampered by the use of non-commercial enzymes or crude extracts for isolating protoplasts. *Dictyopteris pacifica* is part of a brown algal genus well known by its wide chemical diversity and biological properties. *Scytosiphon lomentaria* is an edible brown seaweed with antioxidant, antitumor, and antiviral properties. So far, there are no protoplast isolation protocols using commercial enzymes for these two economic brown algae. In this study, we obtained protoplasts from cultured samples of *D*. *pacifica* and *S*. *lomentaria* using commercially available enzymes. Additionally, we investigated the effects of Driselase inclusion and Ca-chelation pre-treatment on protoplast yields in order to optimize the conditions for protoplast preparations.

**Results:**

Protoplasts were isolated from *Dictyopteris pacifica* and *Scytosiphon lomentaria* using the commercially available Cellulase Onozuka RS (1%) and Alginate lyase (4 U mL^−1^), and short incubation time (4 h). Driselase did not show significant effects on protoplast production in both species. Ca-chelation pre-treatment only increased the number of protoplasts in *D*. *pacifica*. Under optimal conditions, the protoplast yields from *D*. *pacifica* and *S*. *lomentaria* were 4.83 ± 2.08 and 74.64 ± 32.49 × 10^6^ protoplasts g^−1^ fresh weight, respectively. The values obtained for *S*. *lomentaria* were 2–3 orders of magnitude higher than previously reported.

**Conclusions:**

Our results show that high protoplast yields can be obtained from *D*. *pacifica* and *S*. *lomentaria* using a simple mixture of commercial enzymes (Cellulase RS and Alginate lyase) and short incubation time (4 h). This work also represents the first report of protoplast isolation in *D*. *pacifica*. The method proposed here can help to expand protoplast technology in more brown algal species.

## Background

Protoplasts are cells whose cell walls have been removed mostly by enzymatic methods. Although they are usually produced from plant cells, protoplasts can also be obtained from bacteria [1] and fungi [2]. Protoplasts represent a powerful experimental material for in vivo manipulations, offering a wide range of applications for basic and applied research, including genome-editing and gene silencing techniques, and crop improvement bypassing sexual reproduction from species of economic importance [3–5]. The success of this technology relies on reproducible protocols for protoplast isolation, which are mainly achieved by using commercial enzymes [6, 7].

Brown algae are a diverse group of photosynthetic organisms. They are mainly distributed in marine environments and encompass species that are used in human food, animal feed, and traditional medicine [8, 9]. Protoplast isolation has been reported in 33 species of marine brown algae [4, 10–12], including some commercial species like *Undaria pinnatifida* (Harvey) Suringar and *Saccharina japonica* (Areschoug) C.E.Lane, C.Mayes, Druehl & G.W.Saunders [4]. However, protoplast production in other economic brown algae has remained largely unexplored as works on this topic have become scarce during the last decade. In addition, most protocols rely on non-commercial enzymes or crude extracts for protoplast isolation, which make them expensive, time consuming and low reproducible [6, 7].

*Dictyopteris pacifica* (Yendo) I.K.Hwang, H.-S.Kim & W.J.Lee is an epilithic brown seaweed with a complanate and subdichotomous thallus with a matted rhizoidal holdfast. It is endemic from the coasts of Korea and Japan [13]. Studies on vitamins and related substances have shown that this species contains nicotinic and folic acid that play a key role in human energy metabolism [14, 15] and lipoic acid might serve as ancillary treatment for many diseases, such as diabetes and cancer [14, 16]. Also, *D*. *pacifica* is part of a brown algal genus well known by its wide chemical diversity and biological properties [17]. So far, there has not been reports on protoplast isolation in this species.

*Scytosiphon lomentaria* (Lyngbye) Link is tubular-shaped brown algal species distributed in temperate seas worldwide [18]. It is considered a traditional food in China, Taiwan, western coast of Japan and southern coast of Korea. This important economic seaweed has a delicious taste and high nutritional value [19]. This alga is variably consumed as dried food in Japan, boiled soup in China, and mixed food with pork in Taiwan [18, 20, 21]. In addition, *S*. *lomentaria* has shown promising biomedical applications [21, 22]. Protoplast isolation in *S*. *lomentaria* has been accomplished once using a complex mixture of commercial enzymes and a crude extract from the marine herbivore *Trochus maculatus* Linnaeus [23].

This research aims to develop a protocol for protoplast production from *Dictyopteris pacifica* and *Scytosiphon lomentaria* as an effort to expand protoplast technology in commercial brown algae. We isolated the protoplasts from laboratory culture samples using a simple mixture of commercial enzymes. The reason for choosing cultured thalli was that they offer an advantage over field samples in terms of applicability, as the former meets the need for protoplast transformation regarding genetic background [24]. So far, protoplast isolation in *S*. *lomentaria* and other *Dictyopteris* species has been performed on field materials [23, 25, 26]. In addition, we tested the effect of Driselase inclusion and Ca-chelation pre-treatment on protoplast production to determine the best conditions for this process.

## Methods

### Seaweed collection and culture

*Dictyopteris pacifica* was collected at Uljin, Gyeongsangbuk-do, Korea. Representative voucher specimen (MBRB0013TC19281C1, =CUK19281) examined in this study was deposited in the herbarium. Monosporangial germlines were easily detached from old sporophytes in the laboratory and cultured in 60 × 15 mm Petri dishes containing Provasoli-enriched seawater (PES) medium under 14:10-h light/dark photoperiod at 20 °C with light intensity of 40 μmol photons m^−2^ s^−1^ of white LED (DyneBioCo. Korea). To avoid further diatom contamination, GeO_2_ at a final concentration of 6 mg L^−1^ was added to the culture media. Monosporangial germlines produced filaments. Filaments were transferred to 1-L flat-bottomed round flasks filled with 1 L PES medium, under aeration, with a light intensity of 40–72 μmol photons m^−2^ s^−1^ of white fluorescent light. The temperature and photoperiod were the same as indicated above.

*Scytosiphon lomentaria* was collected at Gijang, Busan, Korea. Representative voucher specimen (MBRB0045TC19303C1, =CUK19303) examined in this study was deposited in the herbarium. For culture, thallus was cleaned with sterile seawater in the laboratory, and it started to release spores after 10 min. These were isolated using the hanging-drop technique and then transferred to a 60 × 15 mm Petri dishes containing PES medium under the same conditions for *Dictyopteris pacifica*.

### Identification of the samples

Taxonomic identification was performed using morphological characters according Boo [18], and Lee and Hwang [13], and confirmed molecularly. Genomic DNA extraction, PCR amplification, DNA purification, and sequencing were performed according to Bustamante et al. [27] using cultured samples. The plastid *rbc*L was amplified using the primer combinations described by Kogame et al. [28]. The amplified gene sequences were compared to the GenBank nucleotide database using the BLAST program [29].

### Protoplast isolation

The commercially available cell wall lytic enzymes used for this study included Cellulase Onozuka RS (Yakult Co. Ltd., Japan), Alginate lyase, and Driselase™ *Basidiomycetes* sp. (Sigma-Aldrich, USA). The combinations and conditions for enzymes are shown in Table 1.

**Table 1:** Combinations and concentrations of enzyme mixtures for protoplast isolation from *Dictyopteris pacifica* and *Scytosiphon lomentaria*

Commercial enzymes	Composition of enzyme mixtures			
	A	B	C	D
**Cellulase Onozuka RS (%)**	1	1	1	1
**Alginate lyase (U mL**^**-1**^**)**	4	4	4	4
**Driselase (%)**	1	–	1	–
**Chelation pre-treatment**	No	No	Yes	Yes

Protoplast isolation was carried out as previously described protocol by Coelho et al. [30] with some modifications. Briefly, approximately 100–300 mg of 4–6 mm^2^ explants from cultured foliose thalli of *Dictyopteris pacifica* (4–5 cm in length) or macrothalli of *Scytosiphon lomentaria* (2–3 cm in length) were incubated in a 0.22-μm filter-sterilized enzymatic solution at 20 °C, pH 6 with shaking at 70 rpm in the dark. The solution consisted of 400 mM NaCl, 130 mM MgCl_2_·6H_2_O, 22 mM MgSO_4_, 160 mM KCl, 2 mM CaCl_2_, and 10 mM MES, containing Cellulase Onozuka RS and Alginate lyase, either with or without Driselase. The osmolarity of the enzymatic solution was 1570 mOsm L^−1^ H_2_O. After preliminary experiments, incubation time was set at 4 h for both species.

The inclusion of Driselase was assessed together with Ca-chelation pre-treatment, which was conducted with a calcium-chelating solution [665 mM NaCl, 30 mM MgCl_2_·6H_2_O, 30 mM MgSO_4_, 20 mM KCl, and 20 mM ethylene glycol-bis(β-amino-ethyl ether)-*N*,*N*,*N*′,*N*′- ethylene glycol-bis(β-amino-salt (EGTA-Na_4_) as the calcium chelator; pH5.5] for 20 min prior to enzymatic digestion [30]. Protoplast isolation was repeated four times in each treatment.

Protoplast purification was performed according to Avila-Peltroche and Won [12]. Protoplast yield was estimated by using a hemocytometer (Marienfeld, Germany) and expressed as protoplasts g^−1^ fresh weight (FW). Protoplast size was calculated by using ImageJ 1.46r software (NIH, Bethesda, MD, USA) based on 100 cell measurements for each repetition.

### Viability and cell wall removal

The viability of protoplasts and cell wall removal were assessed by the red chlorophyll autofluorescence and staining with calcofluor white M2R (Sigma-Aldrich, USA), respectively, as previously described [11]. Protoplast viability was further confirmed by using 2.4 μM fluorescein diacetate (FDA; Sigma, USA) and observed under a Leica DMi8 inverted microscope equipped with a 540/46 nm emission filter and a 590 nm suppression filter.

### Statistical analysis

Normality and homoscedasticity were examined by using the Shapiro-Wilk and Levene tests, respectively, prior to conducting parametric tests. Two-way analysis of variance (ANOVA) was used for the comparison of protoplast yield under Driselase inclusion and Ca-chelation pre-treatment. Effect sizes were presented as *ω*^2^ [31] in case of significant results. All these analyses were performed using “car” [32] and “userfriendlyscience” [33] packages in R [34].

Tukey’s post hoc test was used when the results were significant. Post-hoc comparisons were conducted using “multcomp” [35] or “userfriendlyscience” [33] packages in R. For statistical analyses, the significance threshold was set at *p* = 0.01 in order to reduce the true type I error rate (at least 7%, but typically close to 15%) [36]. All graphs were created in Graphpad Prism 6.0 (GraphPad Software, USA).

## Results

### Identification

**Fig. 1:**
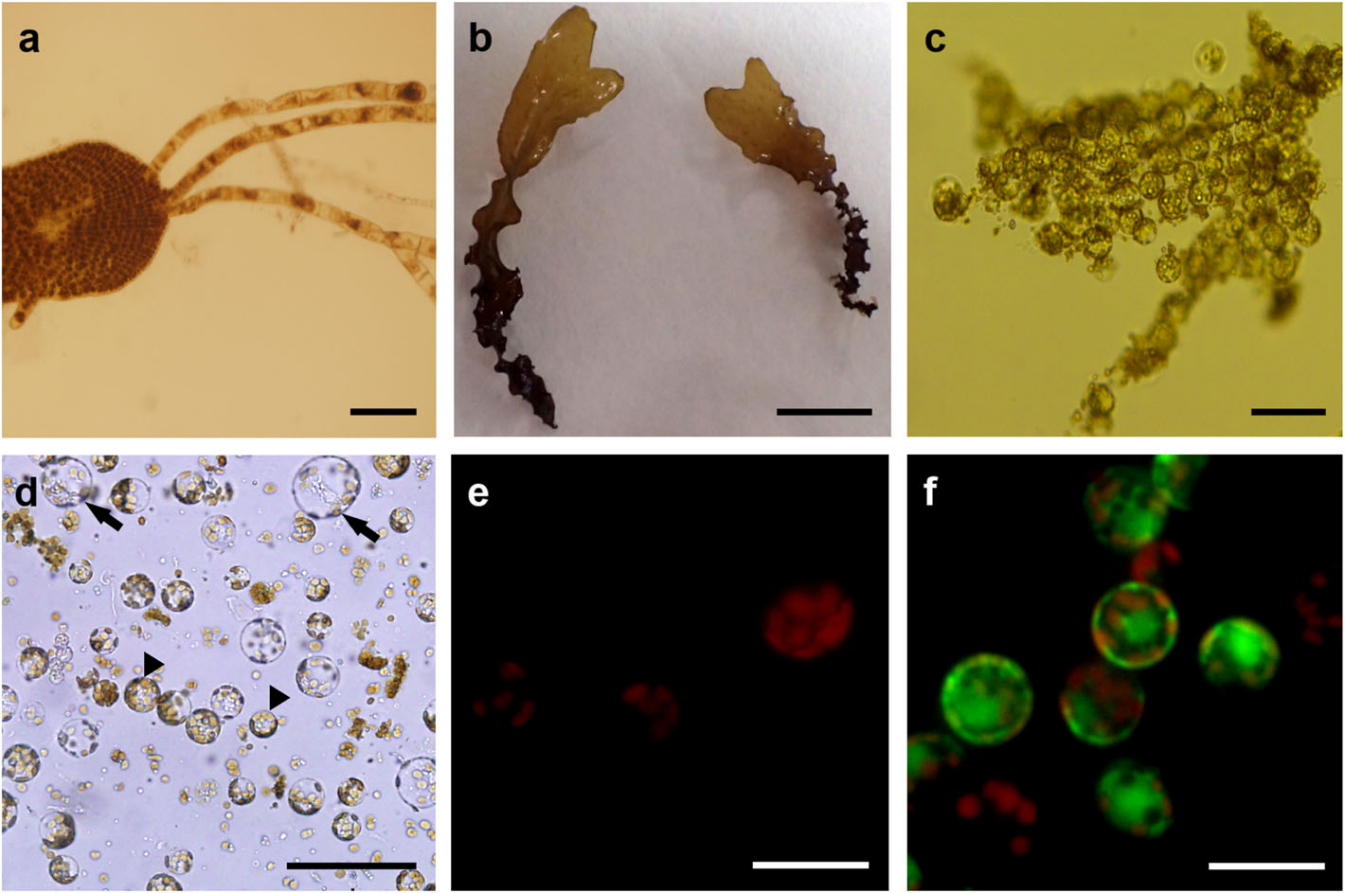
Protoplast isolation from *Dictyopteris pacifica*. **a** A germline blade producing filaments in cultures without aeration (bar = 200 μm). **b** Foliose thalli after 1 month in suspension culture (bar = 1 cm). **c** Spherical protoplasts from blade after 2 h of enzymatic digestion (bar = 100 μm). **d** Freshly isolated protoplasts produced from the cortex (arrowheads) and medulla (arrows) (bar = 100 μm). **e** True protoplasts with red chlorophyll autofluorescence under inverted fluorescence microscope showing no cell walls (bar = 10 μm). **f** Protoplasts with green fluorescence stained with FDA showing their viability (bar = 10 μm)

In our cultures, monosporangial germlines from *Dictyopteris pacifica* produced filaments in petri dishes after 2–3 weeks (Fig. 1a). Filaments developed foliose thalli with subdichotomous blades of 4–5 cm in length in 1 L flask after 1 month and 1 week (Fig. 1b). The blades had the weak midrib, a key character for this species [13]. Our morphological identification of *D*. *pacifica* was confirmed by molecular analysis. The *rbc*L region (1300-bp) (MW715817) of our strain was 100% identical to field samples of *D*. *pacifica* from Korea and Japan [37].

**Fig. 2:**
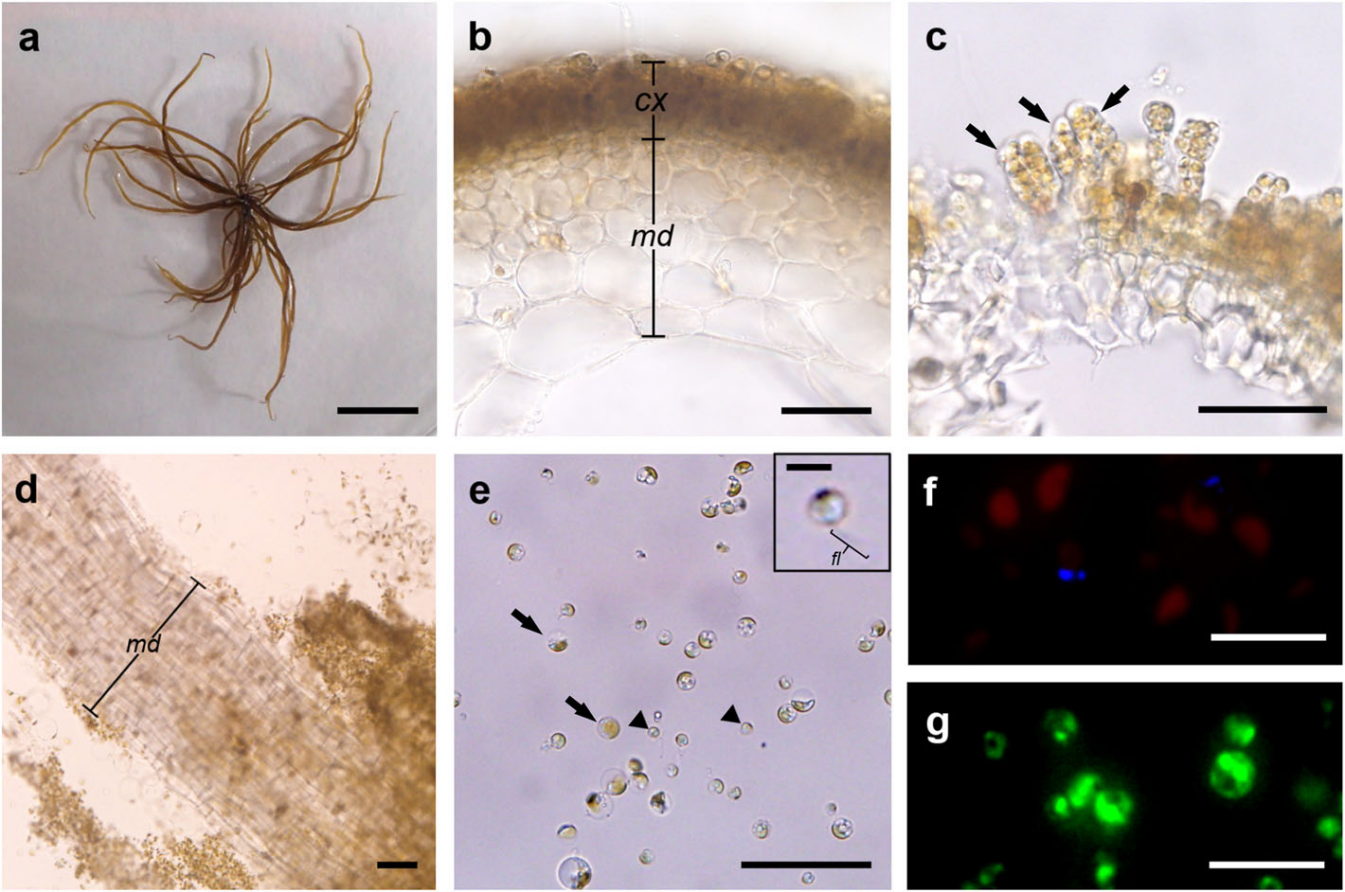
Protoplast isolation from *Scytosiphon lomentaria*. **a** Mature macrothalli after 2 months in suspension culture (bar = 1 cm). **b** Cross section view of macrothallus showing cortex (*cx*) and medulla (*md*) (bar = 50 μm). **c** Plurilocular sporangia (arrows) from a mature macrothallus (bar = 50 μm). **d** Thallus under enzymatic process showing undigested medulla (*md*) (bar = 100 μm). **e** Freshly isolated protoplasts (arrows) from somatic cells and zooids (arrowheads) with flagellum (*fl*) from digested plurilocular sporangia (bar = 50 μm; bar in inset = 5 μm). **f** True protoplasts with red chlorophyll autofluorescence under inverted fluorescence microscope showing no cell walls (bar = 10 μm). **g** Protoplasts with green fluorescence stained with FDA showing their viability (bar = 10 μm)

Spores of *Scytosiphon lomentaria* produced prostrate filamentous thalli. Tubular macrothalli of about 2–3 cm in length were formed in l L flask after 2 months in culture (Fig. 2a, b). They were matched with the description provided by Womersley [38]. Although the thalli did not show marked constrictions as reported by Boo [18], molecular analysis using a *rbc*L region (1342-bp) (MW715818) of our strain was matched with 99.85% to field samples of *S*. *lomentaria* from Korea and Japan [39].

### Protoplast isolation

Protoplast yields from *Dictyopteris pacifica* ranged from 1 to 5 × 10^6^ protoplasts g^−1^ FW (Table 2). Mixture C (Cellulase RS, Alginate lyase, and Driselase with Ca-chelation pre-treatment) and D (Cellulase RS and Alginate lyase with Ca-chelation pre-treatment) produced the large number of protoplasts (Table 2). Two-way ANOVA revealed that pre-treatment had a significant effect on protoplast production (*p* < 0.001; *ω*^2^ = 0.63). Explants pre-treated with Ca-chelation solution reported about 3 times more protoplasts than those ones without pre-treatment. The inclusion of Driselase did not improve protoplast yields (*p* = 0.573). The interaction between both factors did not have a significant effect (*p* = 0.587). Numerous protoplasts were isolated from the cortex and medulla of the blades. Protoplasts were pale yellow-brown to dark brown, spherical shape with several discoid chloroplast (Fig. 1c, d). They were 26.59 ± 5.90 μm (range, 13–52 μm) in diameter. Percentages of true protoplast were 99–100% with calcofluor white staining. The viability of freshly isolated protoplasts was 98–100% with red chlorophyll autofluorescence, and 70% with FDA staining (Fig. 1e, f).

**Table 2:** Protoplast yield of *Dictyopteris pacifica* and *Scytosiphon lomentaria* obtained from different enzyme combinations

Enzyme mixtures	Protoplast yield (× 10^**6**^ protoplasts g^**−1**^ fresh weight)	
	***Dictyopteris pacifica***	***Scytosiphon lomentaria***
**A**	1.98 ± 0.53^a^	65.54 ± 18.69^a^
**B**	1.25 ± 0.67^a^	74.64 ± 32.49_a_
**C**	4.85 ± 1.21^b^	76.54 ± 20.27^a^
**D**	4.83 ± 2.08^b^	57.82 ± 11.39^a^

Superscript letters indicate significant differences among enzyme mixtures for each species (*p* < 0.01). Values are presented as mean ± SD (*n* = 4)

**Fig. 3:**
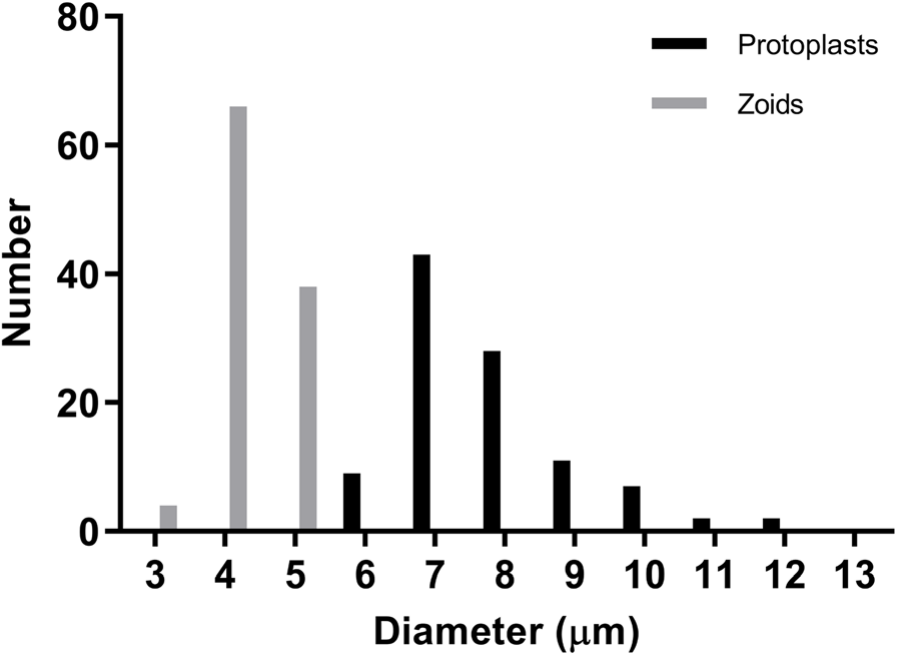
Distribution of the size of somatic cell protoplasts and zooids from *Scytosiphon lomentaria*

Protoplast yields from *Scytosiphon lomentaria* ranged from 58 to 77 × 10^6^ protoplasts g^−1^ FW (Table 2). Mixture C (Cellulase RS, Alginate lyase, and Driselase with Ca-chelation pre-treatment) and B (Cellulase RS and Alginate lyase without Ca-chelation pre-treatment) produced the large number of protoplasts (Table 2). Two-way ANOVA revealed that pre-treatment did not improve protoplast production (*p* = 0.664), while the inclusion of Driselase was not critical for enhancing protoplast yields (*p* = 0.548). After enzymatic digestion, two distinct populations were found with different sizes: protoplasts originating from vegetative cells of the cortex, and more or less mature zooids from digested plurilocular sporangia (Fig. 2c, e; Fig. 3). Medulla were mostly undigested (Fig. 2d). Protoplasts were pale yellow-brown, spherical shape with a single discoid chloroplast. Percentages of true protoplast were 99–100% with calcofluor white staining. The viability of freshly isolated protoplasts was 98–100% with red chlorophyll autofluorescence and 83% with FDA staining (Fig. 2f, g).

## Discussion

In this study, we report the production of high yields of viable and true protoplasts from two economic brown algal species, *Dictyopteris pacifica* and *Scytosiphon lomentaria*, using a simple mixture of commercial enzymes.

For first time, we obtained protoplasts from *Dictyopteris pacifica* in large numbers (1–5 × 10^6^ protoplasts g^−1^ FW) using commercial Cellulase RS and Alginate lyase. Our values were inferior to protoplast yields from *D*. *prolifera* (Okamura) Okamura (3.3 × 10^7^ protoplasts g^−1^ FW) [26] and *D*. *undulata* Holmes (8.2 × 10^6^ protoplasts g^−1^ FW) [25]. However, in these species, protoplasts were produced using a mixture of commercial enzymes and crude extracts from marine herbivores. Ca-chelation pre-treatment was necessary for improving protoplasts yields in *D*. *pacifica*. This positive effect has been also reported in Ectocarpales [30, 40] and Laminariales [41].

Protoplasts from *Scytosiphon lomentaria* were produced in high numbers (5.8–7.7 × 10^7^ protoplasts g^−1^ FW) using a simple enzyme combination of Cellulase RS and Alginate lyase in a single-step method (without pre-treatment). Yamaguchi et al*.* [23] isolated 10^4^–10^5^ protoplasts g^−1^ FW from *S*. *lomentaria* using a complex mixture of commercial enzymes and hepatopancreas extract from the marine herbivore *Trochus maculatus*. Our protocol produced 2–3 orders of magnitude more protoplasts than this previous report. The medullary layer of *S*. *lomentaria* remained largely undigested, suggesting a different composition of cell wall among cell types. For instance, in brown seaweed *Saccharina japonica*, epidermal cell wall differs from cortical cell wall in crystallinity of cellulose and its content [7]. Another reason behind the medullary layer resistance to wall breakdown might be the size (4–6 mm^2^) of explants used in this study. Salvador and Serrano [42] obtained medullary and axial cell protoplasts from the red seaweed *Kappaphycus alvarezii* (Doty) L.M. Liao by using explants less than 1 mm^2^. According to these authors, the small size of the fragments increased the surface area in contact with the enzyme mixture and exposed the medullary and axial zones.

As we used mature thalli, zooids were also isolated from plurilocular sporangia. However, they were easily distinguished based on its size, as they were smaller than protoplasts. This difference was also pointed out by Mejjad et al. [40] when isolating protoplasts and zooids from the filamentous brown alga *Pylaiella littoralis* (Linnaeus) Kjellman.

Despite the presence of mixed-linked glucan (MLG) in brown algal cell walls [43], the use of Driselase, a natural enzyme mixture that degrades MLG [44], did not improve protoplast yields in our experiments. Inoue et al. [7] suggested that at least three components were essential for protoplast isolation from *Saccharina japonica*: cellulase, alginate lyase, and protease. Our results in *Scytosiphon lomentaria* and *Dictyopteris pacifica* suggest that only two components, cellulase and alginate lyase, are sufficient for protoplast production in brown algae. This is consistent with the findings of previous works on filamentous brown algae [11, 12, 45].

In this study, a simple combination of commercial enzymes is essential for establishing reproducible protocols for protoplast isolation. For example, in green marine macroalgae, a simple protocol using only Cellulase Onozuka R-10 has been recently established for consistent production of large amounts of protoplasts [46]. Similarly, commercially available cellulase, macerozyme, and/or pectinase are routinely used for protoplast isolation in higher plants [47]. The effects of protease inhibitors or protein substrates for inactivating toxic components in the enzyme mixtures were not tested in this work. However, these were not an impediment for obtaining large number of protoplasts. Chen and Shyu [48] suggested that these chemicals might not be necessary when incubation time is short, such as the case in this study. They also pointed out that short digestion times allowed protoplast harvesting before being adversely affected by proteases in the enzyme mixture.

## Conclusions

Our work proposes a simple method for isolating protoplasts from *S*. *lomentaria* and it represents the first report of protoplast production from *D*. *pacifica*. In both species, a simple mixture of commercial enzymes (Cellulase RS and Alginate lyase) and short incubation time (4 h) produced high amount of true and viable protoplasts. In *D*. *pacifica*, Ca-chelation pre-treatment was critical for improving protoplast production. These findings can help to develop standard protocols for protoplast technology in brown algae.

## Data Availability

The data that support the findings of this study are available from the corresponding author, Tae Oh Cho, upon reasonable request.

## Abbreviations

FDA: Fluorescein diacetate
FW: Fresh weight
PES: Provasoli Enriched Seawater
